# Comparative Genomics of Shiga Toxin-Producing Escherichia coli Strains Isolated from Pediatric Patients with and without Hemolytic Uremic Syndrome from 2000 to 2016 in Finland

**DOI:** 10.1128/spectrum.00660-22

**Published:** 2022-06-22

**Authors:** Xiangning Bai, Elisa Ylinen, Ji Zhang, Saara Salmenlinna, Jani Halkilahti, Harri Saxen, Aswathy Narayanan, Timo Jahnukainen, Andreas Matussek

**Affiliations:** a Department of Microbiology, Division of Laboratory Medicine, Oslo University Hospital, Oslo, Norway; b Division of Clinical Microbiology, Department of Laboratory Medicine, Karolinska Institute t, Stockholm, Sweden; c Department of Pediatric Nephrology and Transplantation, New Children’s Hospital, University of Helsinki and Helsinki University Hospital, Helsinki, Finland; d Diagnostic and Surveillance Services, Biosecurity New Zealand, Ministry for Primary Industries, Auckland, New Zealand; e Department of Health Security, Finnish Institute for Health and Welfare, Helsinki, Finland; f Division of Infectious Diseases, Department of Medicine Huddinge, Karolinska Institute t, Stockholm, Sweden; g Department of Microbiology, Division of Laboratory Medicine, Institute of Clinical Medicine, University of Oslo, Oslo, Norway; h Laboratory Medicine, Jönköping Region County, Department of Clinical and Experimental Medicine, Linköping University, Jönköping, Sweden; Institut National de Santé Publique du Québec

**Keywords:** Shiga toxin-producing *Escherichia coli*, hemolytic uremic syndrome, pediatric patients, renal outcome, whole-genome sequencing

## Abstract

Shiga toxin-producing Escherichia coli (STEC) infection can cause mild to severe illness, such as nonbloody or bloody diarrhea, and the fatal hemolytic uremic syndrome (HUS). The molecular mechanism underlying the variable pathogenicity of STEC infection is not fully defined so far. Here, we performed a comparative genomics study on a large collection of clinical STEC strains collected from STEC-infected pediatric patients with and without HUS in Finland over a 16-year period, aiming to identify the bacterial genetic factors that can predict the risk to cause HUS and poor renal outcome. Of 240 STEC strains included in this study, 52 (21.7%) were from pediatric patients with HUS. Serotype O157:H7 was the main cause of HUS, and Shiga toxin gene subtype *stx2a* was significantly associated with HUS. Comparative genomics and pangenome-wide association studies identified a number of virulence and accessory genes overrepresented in HUS-associated STEC compared to non-HUS STEC strains, including genes encoding cytolethal distending toxins, type III secretion system effectors, adherence factors, etc. No virulence or accessory gene was significantly associated with risk factors for poor renal outcome among HUS patients assessed in this study, including need for and duration of dialysis, presence and duration of anuria, and leukocyte counts. Whole-genome phylogeny and multiple-correspondence analysis of pangenomes could not separate HUS STEC from non-HUS STEC strains, suggesting that STEC strains with diverse genetic backgrounds may independently acquire genetic elements that determine their varied pathogenicity. Our findings indicate that nonbacterial factors, i.e., characteristics of the host immunity, might affect STEC virulence and clinical outcomes.

**IMPORTANCE** Shiga toxin-producing Escherichia coli (STEC) is a serious public health burden worldwide which causes outbreaks of gastrointestinal diseases and the fatal hemolytic uremic syndrome (HUS) characterized by the triad of mechanical hemolytic anemia, thrombocytopenia, and acute renal failure. Understanding the mechanism underlying the disease severity and patient outcome is of high importance. Using comparative genomics on a large collection of clinical STEC strains from STEC-infected patients with and without HUS, our study provides a reference of STEC genetic factors/variants that can be used as predictors of the development of HUS, which will aid risk assessment at the early stage of STEC infection. Additionally, our findings suggest that nonbacterial factors may play a primary role in the renal outcome in STEC-infected patients with HUS; further studies are needed to validate this.

## INTRODUCTION

Shiga toxin-producing Escherichia coli (STEC) is a genetically and phenotypically diverse group of E. coli strains characterized by the production of one or two different types of Shiga toxin (Stx) (1). STEC infection is the most common cause of the life-threatening hemolytic uremic syndrome (HUS) defined by the triad of mechanical hemolytic anemia, thrombocytopenia, and acute renal failure, which has been observed in 5 to 15% of STEC infection cases (2). STEC infection may also result in asymptomatic carriage and other mild to severe gastrointestinal illness, such as nonbloody or bloody diarrhea (BD) (2). The mechanism underlying the variable pathogenicity is still not fully defined. O157:H7 is the most predominant serotype associated with severe diseases such as HUS (3). In recent years, non-O157 serogroups have been increasingly recognized to cause mild to severe diseases (4–6). The most predominant non-O157 serogroups causing human infections are O26, O45, O103, O111, O121, and O145, referred to as the “big six” (7).

Stx is the primary virulence factor of STEC, which is classified into two immunologically distinct types, i.e., Stx1 and Stx2 (8). Stx1/Stx2 can be further divided into various subtypes, among which Stx2a, Stx2c, and Stx2d are significantly associated with development of HUS, whereas other Stx1/Stx2 subtypes are linked to mild symptoms (9). Besides Stx, other virulence factors also play a role in STEC pathogenicity. Intimin, encoded by *eae* gene, residing on the locus of enterocyte effacement (LEE) pathogenicity island, plays a crucial role in intestinal colonization. The LEE island encodes a type III secretion system (TTSS) which is responsible for the attaching-and-effacing (A/E) lesions on intestinal epithelia (10). STEC strains carrying both *stx2* and *eae* pose a higher risk of triggering severe clinical outcome (11). Moreover, STEC strains harbor additional genes encoding toxins and adherence factors that affect their pathogenic potential, e.g., *ehxA* (enterohemolysin), *astA* (enteroaggregative E. coli heat-stable toxin 1), *cdt* (cytolethal distending toxin), and *lpf* (long polar fimbriae) (12–14). Further studies are warranted to examine the potential role of other genetic factors, including virulence genes, in the development of HUS and severe clinical outcome.

We collected STEC strains from STEC-infected pediatric patients (<17 years of age) with and without HUS from 2000 to 2016 in Finland. In this patient group, age under 3 years, higher leukocyte count and need for dialysis were predictive factors for poor renal outcome among HUS patients, and the presence of *stx2* and *stx2a* were risk factors for HUS (15). Further in-depth microbiological study is essential to understand the genomic characteristics of clinical STEC strains in relation to clinical outcomes. In the current study, by using comparative genomics, we aimed to characterize the genomic features of these strains by utilizing the clinical data to identify bacterial genetic factors that could be used to differentiate the potential of STEC strains to cause HUS and poor renal outcome.

## RESULTS

### Prevalence of serotype and *stx* subtype of STEC strains in relation to HUS status.

Of 240 STEC strains included in genomic analysis in this study, 52 were from HUS patients. In total, 41 serotypes were identified in all STEC strains, among which, O157:H7 accounted for the largest proportion (52.5%; 126/240), followed by O26:H11 (10.4%; 25/240), O145:H28 (7.1%; 17/240), O103:H2 (5%; 12/240). Of note, O157:H7 was significantly overrepresented in HUS STEC strains (67.3%; 35/52) compared to non-HUS STEC strains (48.4%; 91/188) (*P* = 0.0186). Six of 126 O157:H7 strains belonged to clade 8, among which two were from patients with HUS. No difference in other serotypes was found between HUS and non-HUS STEC strains (Table 1).

**TABLE 1:** Serotypes and *stx* subtypes of STEC strains in relation to HUS status

Serotype or *stx* subtype	HUS		Non-HUS		*P* value
	No. of strains	Prevalence (%)	No. of strains	Prevalence (%)	
Serotype					
O157:H7	35	67.31	91	48.4	0.0186
O26:H11	6	11.54	19	10.11	0.798
O145:H28	2	3.85	15	7.98	0.5399
O103:H2	0	0	12	6.38	0.0743
O55:H7	2	3.85	6	3.19	0.6848
O121:H19	2	3.85	3	1.6	0.2966
O78:H4	1	1.92	3	1.6	1
O111:H8	0	0	4	2.13	0.5796
O5:H9	0	0	3	1.6	1
O55:H12	0	0	2	1.06	1
O117:H7	0	0	2	1.06	1
O113:H4	0	0	2	1.06	1
O104:H4	0	0	2	1.06	1
Others	4	7.69	24	12.77	0.462

*stx* subtype					
* stx2a*	45	86.54	68	36.17	4.91e−11^*a*^
* stx1a*+*stx2c*	0	0	46	24.47	4.73e−06^*a*^
* stx1a*	1	1.92	44	23.4	0.0001^*a*^
* stx1a*+*stx2a*	2	3.85	8	4.26	1
* stx1c*	1	1.92	6	3.19	1
* stx2c*	2	3.85	5	2.66	0.6469
* stx1c*+*stx2b*	0	0	3	1.6	1
* stx2b*	1	1.92	2	1.06	0.521
* stx1a*+*stx2d*	0	0	2	1.06	1
* stx2e*	0	0	2	1.06	1
* stx1a*+*stx2b*	0	0	1	0.53	1
* stx2g*	0	0	1	0.53	1

a Benjamini-Hochberg-corrected *P* < 0.05.

Twelve *stx* subtypes/combinations were found in 240 STEC strains, among which *stx2a* accounted for the largest proportion (47.1%; 113/240), followed by *stx1a*+*stx2c* (19.2%; 46/240), *stx1a* (18.8%; 45/240), and *stx1a*+*stx2a* (4.2%; 10/240). *stx2a* was significantly more frequent in HUS STEC strains (86.5%) than in non-HUS STEC strains (36.2%) (*P* = 4.73e-06). In contrast, *stx1a* and *stx1a*+*stx2c* were significantly overrepresented in non-HUS STEC strains (Table 1).

### Virulence factors in correlation with HUS status and renal outcomes.

A number of virulence factor genes were identified in STEC strains; the prevalence of 38 virulence genes was different between HUS STEC and non-HUS STEC strains (P < 0.05) (Table 2). Virulence genes that were significantly overrepresented in HUS STEC strains included cytolethal distending toxins-encoding genes *cdtA*, *cdtB*, and *cdtC*; the autotransporter serine protease gene *espP*; and type III secretion system effector-encoding genes (Benjamini-Hochberg-corrected *P* < 0.05). Other genes that were more prevalent in HUS STEC strains included the adherence gene *efa1*, the heat-stable enterotoxin 1 gene *astA*, the cytotoxin gene *toxB*, genes encoding secretion system proteins, etc. (Table 2). We further evaluated the statistical association between virulence genes and risk factors of poor renal outcomes among 52 HUS patients, including need for and duration of dialysis, presence and duration of anuria, leukocyte counts, and age. HUS patients were categorized into groups based on presence and/or median values of these risk factors (see Table S1 in the supplemental material). No statistical association between virulence genes and these factors was found.

**TABLE 2:** Virulence genes significantly different between HUS STEC and non-HUS STEC strains

Gene	Function	No. of positive strains (%)		*P* value
		HUS STEC (*n* = 52)	Non-HUS STEC (*n* = 188)	
*cdtA*	Cytolethal distending toxin A	29 (55.77)	23 (12.23)	4.05e−10^*a*^
*cdtB*	Cytolethal distending toxin B	29 (55.77)	23 (12.23)	4.05e−10^*a*^
*cdtC*	Cytolethal distending toxin C	29 (55.77)	23 (12.23)	4.05e−10^*a*^
*espR4*	Type III secretion system effector EspR4	37 (71.15)	82 (43.62)	0.0005^*a*^
*efa1*	EHEC factor for adherence Efa-1	33 (63.46)	74 (39.36)	0.0026
*astA*	Heat-stable enterotoxin 1 EAST1	37 (71.15)	92 (48.94)	0.0047
*east1*	Enteroaggregative E. coli heat-stable enterotoxin 1	49 (94.23)	147 (78.19)	0.0077
*espFu*	Type III secretion system effector EspFu	7 (13.46)	6 (3.19)	0.0091
*clpV*	Type VI secretion system ATPase ClpV	49 (94.23)	149 (79.26)	0.0123
*espJ*	Type III secretion system effector EspJ	39 (75)	105 (55.85)	0.016
*espO1-2*	Type III secreted effector	46 (88.46)	136 (72.34)	0.017
*espM1*	Type III secretion system effector EspM1	46 (88.46)	136 (72.34)	0.017
*chuV*	ATP-binding hydrophilic protein ChuV	41 (78.85)	115 (61.17)	0.0212
*chuW*	Oxygen independent coproporphyrinogen III oxidase	41 (78.85)	115 (61.17)	0.0212
*tssG*	Type VI secretion system protein TssG	46 (88.46)	139 (73.94)	0.0264
*nleB2*	Type III secretion system effector NleB2	41 (78.85)	117 (62.23)	0.0313
*shuS*	Heme/hemoglobin transport protein ShuS	40 (76.92)	113 (60.11)	0.0335
*shuA*	Outer membrane hemoglobin receptor ShuA	40 (76.92)	113 (60.11)	0.0335
*fha*	Type VI secretion system protein Fha	46 (88.46)	140 (74.47)	0.0384
*espL4*	Type III secretion system effector EspL4	43 (82.69)	126 (67.02)	0.0385
*tssF*	Type VI secretion system protein TssF	46 (88.46)	141 (75)	0.0391
*rhs*	Type VI secretion system protein PAAR family	35 (67.31)	95 (50.53)	0.0405
*aslA*	Putative arylsulfatase	41 (78.85)	119 (63.3)	0.0455
*espB*	Type III secretion system protein EspB	41 (78.85)	119 (63.3)	0.0455
*nleF*	Type III secretion system effector NleF	49 (94.23)	156 (82.98)	0.0458
*stcE*	Metalloprotease StcE	41 (78.85)	120 (63.83)	0.0460
*paa*	Outer membrane adhesin Paa	49 (94.23)	155 (82.45)	0.0462
*chuT*	Periplasmic heme-binding protein ChuT	2 (3.85)	0 (0)	0.0462
*chuU*	Heme permease protein ChuU	40 (76.92)	115 (61.17)	0.0485
*espP*	Autotransporter serine protease EspP	15 (28.85)	110 (58.51)	0.0002^*a*^
*espR3*	Type III secretion system effector EspR3	10 (19.23)	90 (47.87)	0.0002^*a*^
*ospG*	Type III secretion system effector kinase OspG	0 (0)	33 (17.55)	0.0004^*a*^
*toxB*	Cytotoxin ToxB	13 (25)	87 (46.28)	0.0067
*cif*	Type III secretion system effector Cif	8 (15.38)	60 (31.91)	0.0231
*hcp1*	Type VI secretion system protein Hcp family	11 (21.15)	70 (37.23)	0.0319
*nleA*	Non-LEE-encoded effector NleA	2 (3.85)	29 (15.43)	0.0333
*espX4*	Type III secretion system effector EspX4	48 (92.31)	185 (98.4)	0.0414
*entA*	23-dihydroxybenzoate-23-dehydrogenase Ent	0 (0)	15 (7.98)	0.0461

a Benjamini-Hochberg-corrected *P* < 0.05.

### Identification of STEC/ETEC hybrid pathotype.

Three STEC strains carried the heat-stable enterotoxin-encoding gene *sta*, which is a virulence determinant of enterotoxigenic Escherichia coli (ETEC), and were therefore defined as having an STEC/ETEC hybrid pathotype. The three STEC/ETEC strains belonged to the serotypes O100:H2O, O187:H28, and O2:H27 and carried *stx2e*, *stx2g*, *stx2a* subtypes, respectively. The three STEC/ETEC strains were from diarrheal patients without HUS, ages 1, 4, and 7, respectively.

### Whole-genome phylogeny of all STEC strains.

**FIG 1:**
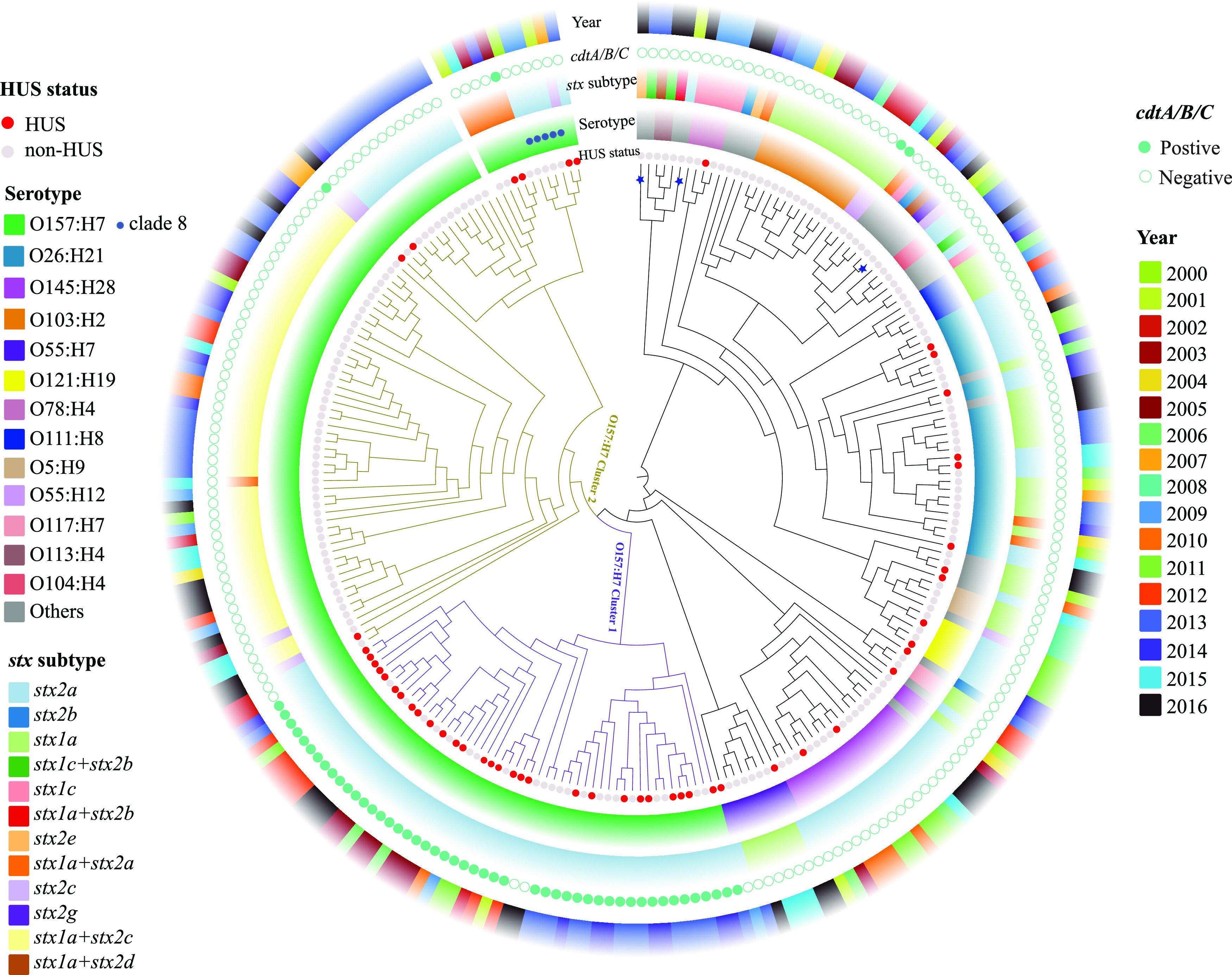
Whole-genome phylogeny of Shiga toxin-producing Escherichia coli (STEC) isolates. Circular representation of the Gubbins phylogenetic tree generated from the concatenated sequences of the shared loci found in the wgMLST analysis. Gubbins tree was annotated with relevant metadata using an online tool ChiPlot (https://www.chiplot.online/). Circles (from inner to outer circle) represent HUS status, serotype (O157:H7 clade 8), *stx* subtype, presence of *cdtA*/*cdtB*/*cdtC* genes, and year of isolation. Three STEC isolates carrying heat-stable enterotoxin encoding gene *sta* are marked with blue stars on corresponding branches. Branch lengths are ignored to better illustrate the two O157:H7 clusters.

A whole-genome phylogenetic tree was constructed by alignment of 2,419 shared genes in 240 STEC strains and the reference genome of O157:H7 strain Sakai (Fig. 1). No distinct phylogenetic cluster was found between HUS STEC and non-HUS STEC strains. O157:H7 strains clustered together, separate from non-O157 STEC isolates, while close to O55:H7 and O145:H28 strains. Two O157:H7 clusters and several subclusters were observed on O157:H7 strains. Most HUS STEC O157:H7 strains were grouped into one cluster, termed O157:H7 cluster 1, consisting of 50 strains. Only 7 of 76 O157:H7 strains within cluster 2 were from patients with HUS. Of note, the six O157:H7 strains belonging to clade 8 clustered together within cluster 2. O157:H7 strains carrying the two predominant *stx* subtypes, *stx2a* and *stx1a+stx2c*, clustered closely based on their *stx* subtypes. It should be noted that all O157:H7 strains carrying *cdtA*, *cdtB*, and *cdtC* genes, which were significantly associated with HUS status, clustered together within O157:H7 cluster 1 with two exceptions. Non-O157 STEC strains with same serotype were more likely to cluster together, e.g., O26:H11 and O145:H28 strains; however, strains associated with HUS were distributed over all the non-O157 phylogenetic clusters. Strains with similar genetic backgrounds were found over years.

### Pangenome-wide association study (PWAS).

**FIG 2:**
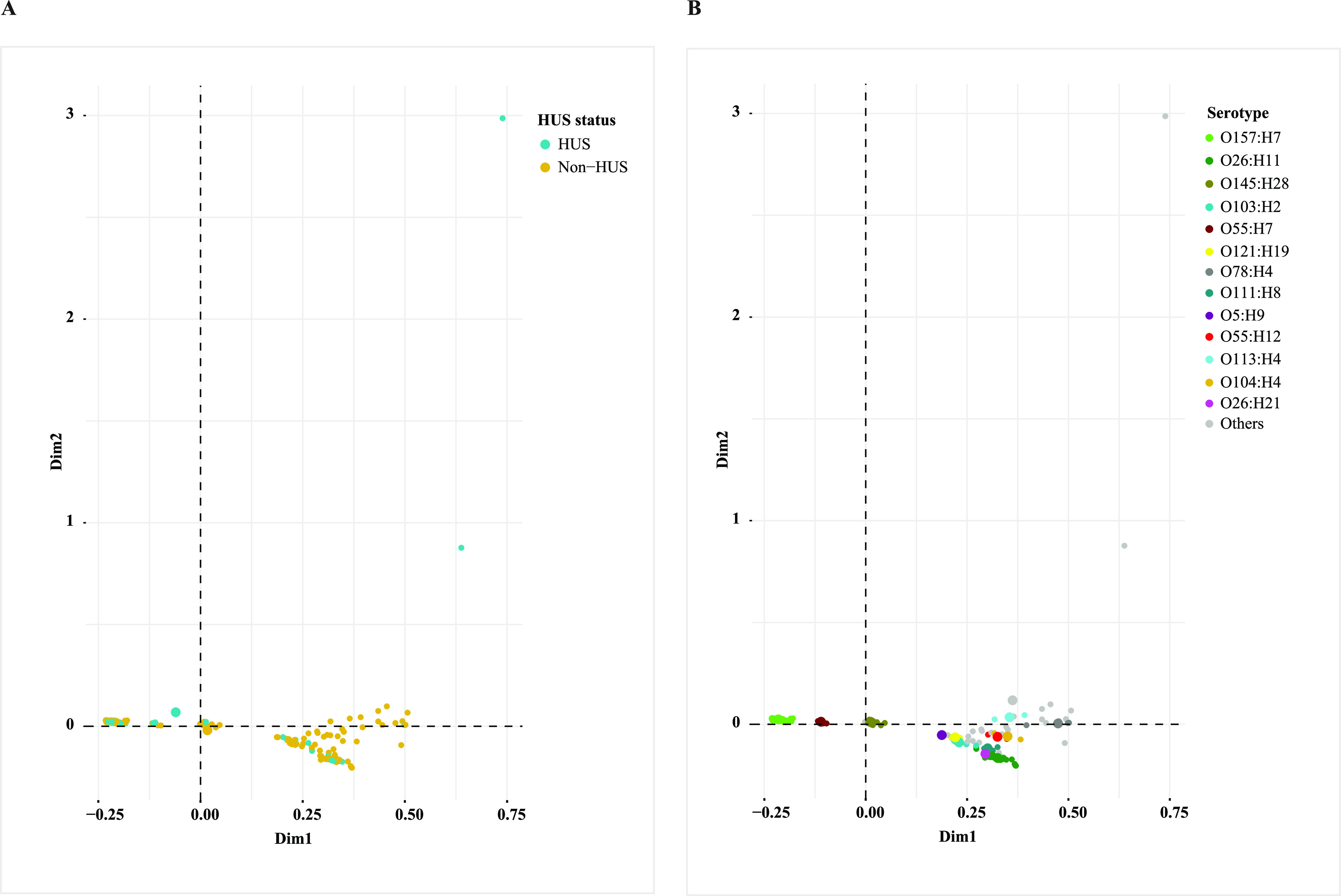
Multiple correspondence analysis plot comparing pangenomes of Shiga toxin-producing Escherichia coli (STEC) isolates in this study. Strains from patients with HUS and non-HUS are indicated by the blue and yellow rings, respectively (A). The main serotypes are marked in different colors (B).

A total of 17,643 genes were identified in the pangenomes of 240 STEC strains using Roary. Scoary identified hundreds of accessory genes that were significantly different between HUS STEC strains and non-HUS STEC strains (Tables S2 and S3), among which 297 genes were significantly overrepresented in HUS STEC strains (Benjamini-Hochberg corrected *P* < 0.05) (Table S2). These genes encoded cytolethal distending toxin, adhesins, transcriptional regulators, phage proteins, etc. A number of genes were related to hypothetical proteins (HP) annotated by Prokka. We did manual BLASTN searching in GenBank of these HP genes and their surrounding genes; many of them are of mobile-element origin, and their functions are poorly characterized. Multiple correspondence analysis (MCA) of pangenomes could not separate HUS STEC strains from non-HUS STEC strains, and O157:H7 strains were separated from non-O157 strains (Fig. 2), similar to whole-genome phylogeny. A PWAS was further performed on 52 HUS STEC strains to identify any accessory gene associated with risk factors for poor renal outcome among HUS patients. Scoary identified a number of accessory genes that were associated with the need for and longer duration of dialysis, presence and longer duration of anuria, and higher leukocyte count; however, most of these genes encode hypothetical proteins, and all associations lost statistical significance after multiple testing correction (data not shown). MCA of pangenomes could not separate HUS STEC strains into subgroups based on clinical variables of HUS patients assessed in this study; O157:H7 HUS STEC strains formed a separate cluster from non-O157 strains by MCA (data not shown).

## DISCUSSION

In the present study, we performed comparative genomic analyses on a large collection of clinical STEC strains from pediatric patients with and without HUS in Finland. Our study showed that O157:H7 was the main cause of HUS in Finland, similar to reports in other countries (16, 17). Manning et al. reported that O157:H7 strains can be classified into nine clades based on SNPs (18), among which clade 8 strains have been reported to produce higher level of Stx2 and pose higher risk of HUS (18, 19). This was endorsed by a recent Swedish study showing that all O157:H7 strains from HUS patients with one exception belonged to clade 8 (16). Interestingly, our data showed that only 2 of 35 O157:H7 strains from Finnish HUS patients belonged to clade 8. This suggests that genetic backbones of highly virulent and predominant O157:H7 strains may differ by geographical locations; this may also apply to other serotypes. In addition to O157:H7, two of the top six non-O157 STEC serotypes, O26:H11 and O145:H28 (20), were found in HUS STEC strains. Other HUS-associated serotypes included O55:H7, O78:H4, O25:H4, etc., indicating the pathogenic potential of some nonpredominant serotypes in Finland.

The most serious manifestation of STEC-related disease, such as HUS, is more often associated with strains that produce Stx2a than other Stx2/Stx1 subtypes (8, 21). Our study showed consistently that the presence of *stx2a* was significantly associated with HUS. Future functional studies are required to investigate the production level of different Stx subtypes in disease progression and to explore the underlying factors regulating Stx production, e.g., prophage regulatory elements. Besides *stx2a*, we observed that cytolethal distending toxin encoding genes *cdtA*, *cdtB*, and *cdtC* were significantly more frequent in HUS STEC strains than in non-HUS STEC strains in Finland. *cdtA*, *cdtB*, and *cdtC* are adjacent or slightly overlapping genes encoding a cytolethal distending toxin (CDT), i.e., CDT-V, which characteristically distends cell morphology and eventually causes cell death (22). It has been shown that in O157:H7 STEC strains, *cdt-V* was significantly more frequent in isolates from patients with diarrhea than in isolates from patients with HUS or asymptomatic carriers (23), while among *eae*-negative non-O157 STEC strains, *cdt*-*V* was significantly more frequent in isolates from patients with HUS or with diarrhea than in isolates from asymptomatic carriers (24). Our data suggest a potential role of cytolethal distending toxins among O157:H7 strains in HUS pathogenesis; however, this finding remains to be validated by further functional studies and data from other geographical locations. Other virulence genes associated with HUS included genes encoding type III secretion system proteins, adherence factors, heat-stable enterotoxin 1, etc. Although we did not find an association between the intimin gene *eae* and HUS status or clinical outcomes, it is evident that different *eae* genotypes are associated with disease severity (25). Future studies are warranted to molecularly and functionally characterize these virulence genes/variants in correlation to STEC-associated disease severity and clinical outcomes.

We further evaluated the potential association between virulence genes and risk factors of poor renal outcomes in HUS patients, including need for and duration of dialysis, presence and duration of anuria, leukocyte counts, and age, as described previously (15). No statistical association between virulence genes and these factors was found. Pangenome-wide association studies identified accessory genes that were overrepresented in the STEC strains from HUS patients with poor renal outcomes; however, most of the genes encode hypothetical proteins, and all associations lost statical significance after multiple testing correction. In addition to virulence genes, e.g., *cdtA*, *cdtB*, and *cdtC*, we identified multiple accessory genes significantly overrepresented in HUS STEC strains compared to non-HUS STEC strains; most genes were of mobile-element origin (e.g., prophage) and hypothetical protein-encoding genes whose functions are poorly characterized. However, none of these significant genes was unique to the HUS STEC group, in line with a previous report from Norway (26). These results highlight the idea that nonbacterial factors, e.g., host immunity, may play an important role in HUS pathogenesis and renal outcome (27).

Whole-genome phylogeny showed that HUS STEC and non-HUS STEC strains did not form a separate phylogenetic cluster, which was in line with previous reports from Sweden and Norway (26, 28). Most HUS-associated O157:H7 strains in this study were distributed in one cluster, which we termed O157:H7 cluster 1. Of note, all O157:H7 strains within this cluster, except two, were positive for *cdtA*, *cdtB*, and *cdtC* genes, which were found to be associated with HUS, while the majority of O157:H7 cluster 2 strains were negative for the three genes and were from patients without HUS. Our results suggest that *cdt* could be considered a predictor of phylogenetic relatedness among O157:H7 strains and a contributor to the development of HUS. It should be noted that this finding may be restricted to Finnish isolates; studies from other geographical locations are essential to confirm this. In line with whole-genome phylogeny, MCA of pangenomes could not separate HUS STEC strains from non-HUS STEC strains either. These findings suggest that different genetic factors may contribute to pathogenic potential in different STEC phylogenetic lineages. Three STEC strains carrying the heat-stable enterotoxin-encoding gene *sta* were distributed separately, indicating the genetic diversity of the STEC/ETEC hybrid. It is notable that serotypes of the three STEC/ETEC isolates (O187:H28, O100:H2O, and O2:H27) have been reported in STEC/ETEC hybrids previously (29–31), and STEC/ETEC hybrids are seemingly more likely to carry uncommon *stx* subtype, e.g., *stx2e* and *stx2g*, which is corroborated by this study (31). Further studies are required to investigate if STEC strains with certain genetic backbones more easily pick up exogenous genes, thereby facilitating the emergence of hybrid pathotypes.

This study has limitations. First, the clinical data for STEC-infected patients without HUS were unavailable, and therefore, we could not evaluate associations between bacterial genetic factors and clinical outcome in patients who did not have HUS but who had other severe symptoms, such as bloody diarrhea. Second, young age was considered a risk factor for development of HUS (32), and all the patients included in this study were below 17 years of age; thus, the findings from the present study may not apply to elderly individuals infected with STEC.

In conclusion, this study characterized the genomic traits of a large collection of clinical STEC strains from pediatric patients in Finland over 16 years. Our study shows that O157:H7 serotype is the main cause of HUS and *stx2a* subtype is significantly associated with HUS. Comparative genomics and pangenome-wide association studies identified a number of virulence and accessory genes that were overrepresented among HUS-associated STEC isolates compared to non-HUS STEC strains; these genes mainly encode cytolethal distending toxin, type III secretion system effectors, adherence factors, etc. No virulence or accessory genes were found to be significantly associated with risk factors for poor renal outcome among HUS patients assessed in this study, suggesting that nonbacterial factors, e.g., characteristics of the host immunity, may play a primary role in renal outcome in patients with HUS. Further research is warranted to validate and expand these findings.

## MATERIALS AND METHODS

### Ethics statement.

The Ethics Committee of the University of Helsinki approved the use of patients’ information and the study protocol (HUS/1274/2017).

### Collection of STEC isolates and clinical data.

STEC isolates were collected from STEC-infected pediatric patients (<17 years of age) in Finland from 2000 to 2016 as described previously (15). The clinical and laboratory data for patients were retrieved from the medical records until the most recent follow-up visit. HUS patients were further categorized into groups based on presence and/or median values of clinical variables that were found to be risk factors for worse renal outcome, including need for and duration of dialysis treatment, presence and length of anuria, leukocyte count, and age, as previously reported (15). The clinical parameters for HUS patients included in this study are shown in Table S1.

### Whole-genome sequencing and assembly.

Whole-genome sequencing (WGS) was performed by using Illumina MiSeq or HiSeq sequencers as previously described (15). The raw sequencing reads were reprocessed to ensure the quality of genomes in subsequent analysis. Briefly, the raw sequencing reads was assessed with FastQC (version 0.11.8) (https://github.com/s-andrews/FastQC). Trimmomatic (version 0.38) was used to trim the adapter sequences, and low-quality bases were trimmed when the average quality score per base dropped below 20 in a 4-base sliding window (33). Sequencing reads that were shorter than 30 bp were eliminated from further analysis. The trimmed reads were *de novo* assembled with SPAdes (version: 3.15.3) with “isolate” option (34). The draft genome sequences were annotated with Prokka (version 1.14.6) (35) using the built-in Escherichia-specific BLAST database. Genomes that had signs of contamination (e.g., abnormal size and GC content) were discarded, and a total of 240 STEC strains were included in this genomic study (Table S1).

### Characterization of *stx* subtypes, serotypes, and virulence factor genes.

The *stx* subtypes of all STEC isolates were determined by ABRicate version 1.0.1 (https://github.com/tseemann/abricate) using default parameters as described previously (16). Briefly, an in-house *stx* subtyping database was created with ABRicate by integrating representative nucleotide sequences of all identified *stx1* and *stx2* subtypes, which included *stx1* and *stx2* subtypes previously reported by Scheutz et al. (36), and several recently reported Stx2 subtypes, i.e., Stx2h (37), Stx2i (38), Stx2j (39), Stx2k (40), and Stx2l (13), and Stx2m (41). The assemblies were then used to search against the *stx* subtyping database. Serotype was determined by comparing assemblies to the SerotypeFinder database (https://cge.food.dtu.dk/services/SerotypeFinder/) using ABRicate version 1.0.1. Fisher’s exact test using R software version 4.1.1 (https://www.r-project.org) was used to assess association between specific *stx* subtypes/serotypes and HUS status; association with a *P* value of <0.05 was regarded as statistically significant.

The VFDB database (http://www.mgc.ac.cn/VFs/) was used for determination of virulence factor genes. The presence/absence of genes was determined using ABRicate version 1.0.1 with default parameters. Statistical association between virulence genes and strain classifications (HUS versus non-HUS; levels of clinical variables among HUS patients) was assessed with Fisher's exact test. Benjamini-Hochberg method in R was used to correct *P* values in the case of multiple testing. Virulence factors with Benjamini-Hochberg-corrected *P* values below 0.05 were considered statistically significant. Whenever no significant association was identified after correction, results for uncorrected analysis were given.

### Determination of clade 8 in O157:H7 strains.

A previous study showed that patients with HUS were significantly more likely to be infected with clade 8 variants of O157:H7 strains (18), suggesting that the clade 8 lineage has acquired critical factors that contribute to more severe disease. The clade 8-specific single-nucleotide polymorphism (SNP) of O157:H7 strain was identified from the genome assemblies with an in-house program (https://github.com/jizhang-nz/clade8) (19).

### Whole-genome phylogenetic analysis.

The phylogenetic relationships of all STEC isolates were assessed by whole-genome multilocus sequence typing (wgMLST) and whole-genome phylogeny analysis. To define wgMLST allelic profiles, we used Fast-GeP (https://github.com/jizhang-nz/fast-GeP) (42) with default settings. The complete genome sequence of O157:H5 strain Sakai (NC_002695.2) was used as a reference. The whole-genome polymorphic site-based phylogeny was inferred from the concatenated sequences of the coding sequences shared by all the whole-genome sequences. All the regions with elevated densities of base substitutions were eliminated and a final maximum-likelihood tree was generated by Gubbins (version 2.3.4) (43) with default settings. The phylogenetic tree was annotated with relevant metadata using on online tool ChiPlot (https://www.chiplot.online/).

### PWAS.

The pangenomes of all STEC isolates were calculated from the harmonized genome annotations produced by Prokka using Roary (https://github.com/sanger-pathogens/Roary) (44) with the following command: roary -s -e –mafft *.gff. Pangenomes consist of a complete set of core and accessory genes in all isolates (45). In this study, core genes are defined as genes present in ≥99% of isolates, and the remaining are termed accessory (noncore) genes. The accessory genes were associated with clinical symptoms (HUS versus non-HUS) using Scoary v1.6.16 (run with 1,000 permutation replicates) (46). Accessory genes were reported as statistically significantly associated with a variable if they attained a Benjamini-Hochberg-corrected *P* value below 0.05. MCA of pangenomes was performed using the gene presence/absence table generated from Roary as previously described (16). The R function MCA from the R package FactoMineR was used for the analysis (47). PWAS was further performed on HUS STEC strains to determine whether any accessory gene was associated with risk factors for poor renal outcome in HUS patients, including need for and duration of dialysis, presence and duration of anuria, and leukocyte counts (15).

### Data availability.

The genome assemblies of 240 STEC strains were submitted to GenBank under the BioProject number PRJNA808114. Details of 240 assemblies are provided in Table S1.
